# Seaweed Polysaccharides: A Rational Approach for Food Safety Studies

**DOI:** 10.3390/md23110412

**Published:** 2025-10-22

**Authors:** João Cotas, Mariana Lourenço, Artur Figueirinha, Ana Valado, Leonel Pereira

**Affiliations:** 1 CFE—Centre for Functional Ecology, Science for People & Planet, Marine Resources, Conservation and Technology—Marine Algae Lab, Department of Life Sciences, University of Coimbra, 3000-456 Coimbra, Portugal; 2 Department of Life Sciences, University of Coimbra, 3000-456 Coimbra, Portugal; 3 University of Coimbra, Faculty of Pharmacy, 3000-548 Coimbra, Portugal; 4 Associated Laboratory for Green Chemistry (LAQV) of the Network of Chemistry and Technology (REQUIMTE), University of Coimbra, 3000-548 Coimbra, Portugal; 5 Higher School of Health Technology (ESTESC), Polytechnic University of Coimbra, Rua da Misericórdia, Lagar dos Cortiços, S. Martinho do Bispo, 3045-093 Coimbra, Portugal; 6 Research Center for Natural Resources, Environment and Society (CERNAS), Polytechnic University of Coimbra, Bencanta, 3045-601 Coimbra, Portugal; 7 H&TRC—Health & Technology Research Center, Coimbra Health School, Polytechnic University of Coimbra, Rua 5 de Outubro, 3045-043 Coimbra, Portugal; 8 MARE—Marine and Environmental Sciences Centre/ARNET—Aquatic Research Network, University of Coimbra, 3000-456 Coimbra, Portugal

**Keywords:** INFOGEST, alginate, agar, carrageenan, digestion, quality control systems

## Abstract

Marine macroalgae (seaweed) are a rich source of bioactive polysaccharides such as agar, carrageenan, and alginate. These three compounds are classified as food additive ingredients, widely used as gelling, thickening, stabilizing, and emulsifying agents in the food, nutraceutical, pharmaceutical, and cosmetic industries. However, the growing concern for a safer world has sparked renewed interest in their safety evaluation. Unlike synthetic compounds with specified structures, seaweed polysaccharides exhibit substantial structural heterogeneity due to variations in species, habitat, and processing, affecting bioactivity, digestibility, and interactions within the gastrointestinal tract. Although the safety of these compounds is generally accepted, there are still significant gaps in our understanding of their physicochemical behaviour. This highlights the need to develop a standardized digestion model to ensure their safety and evaluate their potential long-term health effects. Most of these compounds are only partially absorbed in the upper gastrointestinal tract, where they are fermented into metabolites with varying health effects. The safety of carrageenan, in particular, remains a subject of debate due to ambiguous results reported by various researchers’ groups. This review highlights the importance of adopting standardized digestion assays, integrated analytical tools, and multidisciplinary approaches. These are crucial for thoroughly evaluating the molecular integrity, metabolism, and biological impact of seaweed polysaccharides, which will ultimately support evidence-based regulatory frameworks and ensure their safe use in human nutrition. This critical analysis focuses on food safety and security, with a methodology that can be applied to other foods or compounds.

## 1. Introduction

Seaweed are rich sources of proteins, dietary fibres, essential amino acids, minerals, vitamins, pigments, and fatty acids, which together contribute to their potential as functional foods. In addition to their nutritional value, seaweed-derived compounds exhibit diverse bioactivities, including antioxidants, antihypertensive, antidiabetic, anti-inflammatory, antitumoral, antiviral, and antimicrobial. Recent interest has also grown around their gastronomic applications, as consumer demand for sustainable and health-promoting foods increases. However, challenges such as variability in composition, heavy metal accumulation, and the need for regulatory harmonization remain important considerations for their safe integration into human diets [1].

Marine macroalgae (seaweed) were recently identified as great producers of various bioactive polymers and metabolites with high biochemical importance, resulting in increased curiosity in seaweed research. Polysaccharides, one of the many bioactive ingredients found in seaweed, have been shown to exhibit a wide range of bioactivities, including antioxidants, antibacterial, anticarcinogenic, immune-enhancing, and anti-inflammatory. Given their reported bioactivities, seaweed polysaccharides show considerable potential for a wide range of applications in the food, feed, pharmaceutical, nutraceutical, and cosmetic sectors [2].

The growing demand for natural, sustainable alternatives to synthetic food additives has propelled seaweed-derived polysaccharides to the leading edge of food research and manufacturing. Polysaccharides extracted from red and brown seaweed, such as agar, carrageenan, and alginate, are commonly used in food production due to their gelling, emulsifying, thickening, and stabilizing properties [3,4]. Nevertheless, as their use expands into nutraceutical and functional food applications, a more rigorous, biochemistry-based approach to food safety research becomes essential [5,6,7]. Seaweed polysaccharides are exceptionally rich biopolymers, made up of many different monosaccharides, with high variability in branching patterns, degrees of polymerization, and functional group modifications (such as sulfation and acetylation). Temperature, salinity, and nutrient availability all have an impact on the structural complexity of seaweed. Unlike many synthetic food additives with well-defined molecular structures, seaweed polysaccharides vary significantly from batch to batch [8]. As a result, even goods branded as “agar”, “alginate” or “carrageenan” can vary significantly in content, thereby affecting their bioactivity, digestibility, and possible interactions within the human gastrointestinal system. Even though they are approved by FAO (Food and Agriculture Organization of the United Nations), FDA (Food and Drug Administration) and EFSA (European Food Safety Authority), they need to be evaluated under their physicochemical parameters, although academic research continues to show potential human health problems [3,4,9,10,11,12].

Seaweed polysaccharides are not absorbed in the upper gastrointestinal tract but rather undergo partial enzymatic hydrolysis and microbial fermentation in the colon [13,14]; however, some small fragments such as oligosaccharides, monosaccharides, sulphate esters, and uronic acids, can potentially be absorbed in the upper gastrointestinal tract [11,12,15,16,17]. This breakdown process can generate short-chain fatty acids, which, depending on the structure of the parent compound, can have either beneficial or detrimental impacts on health [14]. Despite this, there is a scarcity of published information on human health outcomes of seaweed polysaccharides. The available articles often lack thorough physicochemical and chemical characterization of the polysaccharides studied, thus creating a significant gap in safety assessments [7,18]. Furthermore, there are no defined in vitro digestion models for these polysaccharides, making it impossible to compare data from different studies or set regulatory criteria.

One of the most difficult issues in regulating seaweed polysaccharides is their ambiguous classification. They are frequently considered as food additives, dietary fibers, or even functional ingredients, depending on their application and chemical profile. This ambiguity highlights the need for multidisciplinary studies to clarify their classification and regulatory status.

This critical review investigates how new methodologies and technologies may improve the safety and security of seaweed food-approved polysaccharides. It focuses on creating a standard paradigm to assess their safety to human health and their usage as food additives. This review focuses on the medical, biomedical and biochemical understanding of the human digestion, as well as the possible pathways through which polysaccharides may interact within the human gastrointestinal tract (positive and negative mechanism of action). Giving an insightful view of this paradigm in the food safety of widely used food emulsifiers additives.

## 2. Seaweed Polysaccharides: Structural and Functional Overview

### 2.1. Chemical Diversity

The chemical structure of seaweed polysaccharides is distinct, depending on the taxonomic group to which they belong, differing according to the species, the season and the extraction method. The colloids authorized in the food industry, and widely used worldwide, are alginate (extracted from brown seaweed), agar and carrageenan (extracted from red seaweed) [19,20,21,22].

#### 2.1.1. Alginate

**Figure 1:**
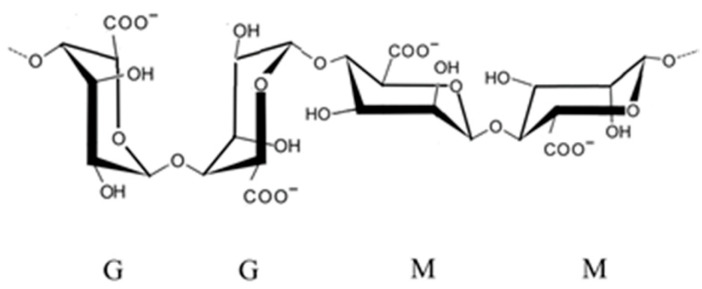
Chemical structure of alginic acid. Caption: G—guluronic acid; M—mannuronic acid.

**Figure 2:**
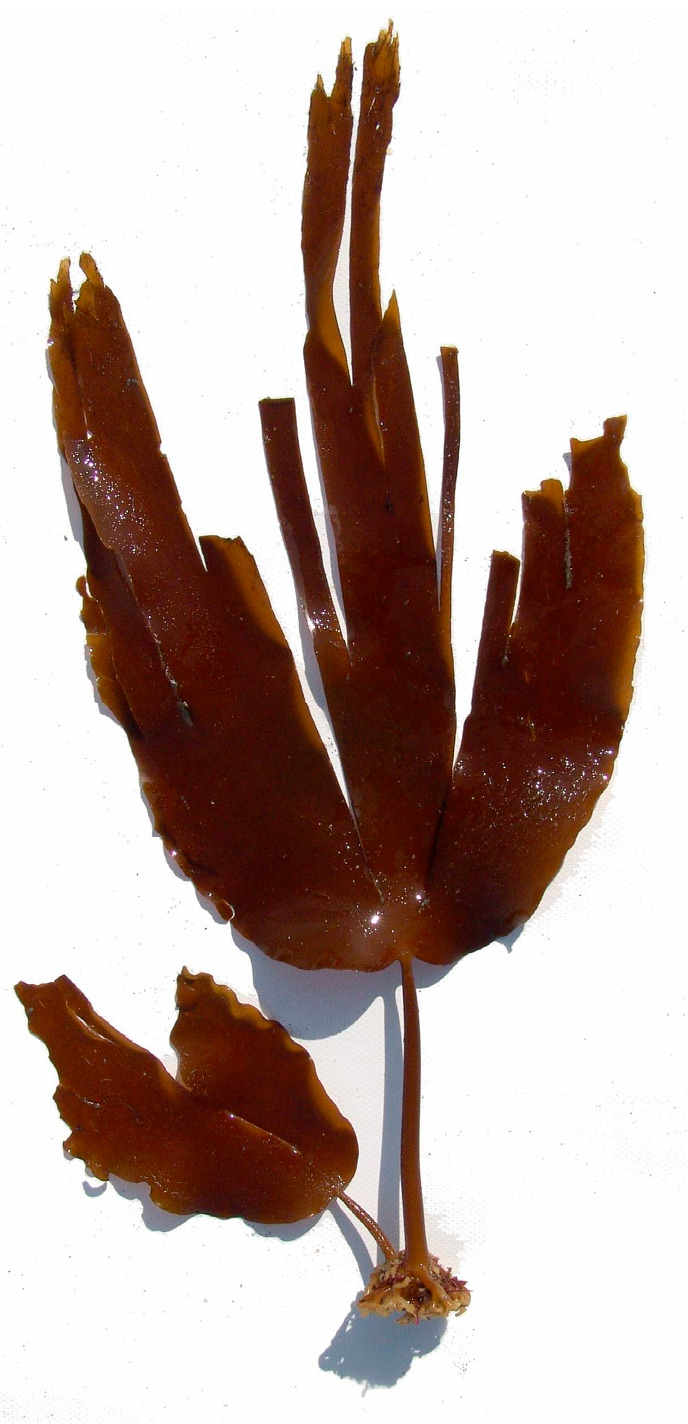
*Laminaria ochroleuca* (Phaeophyceae).

Alginate (Figure 1) is the main polysaccharide of brown seaweed, mainly from kelps (large brown seaweeds) (Figure 2). Various salt forms are approved by the FDA and EFSA. Alginate is an anionic polymer based on β-D-mannuronic acid and 1,4-α-L-guluronic acid monomers [23]. For food certification purposes, the molecular weight of alginate is not considered; however, it is considered for good practices in the extractive industry associated with the food sector, especially in the extraction process [24].

The industrial extraction of this polysaccharide involves several steps: washing the seaweed to remove impurities; pretreatment with heated acid (typically hydrochloric acid for 24 h) to remove pigments, proteins, and lipids [25,26]. Next, solid–liquid extraction occurs, where the solid residue is subjected to an alkaline treatment (sodium carbonate) followed by a centrifugation or filtration process. After this process, hydrochloric acid is added to the liquid extract to precipitate the alginic acid dissolved in the solution, in the form of sodium alginate. After precipitation, the precipitated solution is centrifuged/filtered to obtain only the precipitated alginate. After this step, the alginate is dried and ground for later application [27].

Alginate is classified as a non-organic compound and is approved by the FDA (Table 1) and the EFSA as a food ingredient [28]. In this context, the application and labelling of food products containing alginate are regulated according to the European Union Commission Regulation (1333/2008) as E400 (alginic acid), E401 (sodium alginate), E402 (potassium alginate), E403 (ammonium alginate), E404 (calcium alginate), and E405 (propylene glycol alginate) [24].

The main characteristics of alginate are its high viscosity and strong absorption capabilities. Due to these properties, alginate can be used to thickening food products, such as jellies, marmalades, sauces (e.g., mayonnaise), syrups, and ice cream [29,30]. The FDA approved alginate for human consumption after toxicological testing. However, the FDA requires evidence of good manufacturing practices in alginate extraction and its use at limited concentrations, which vary depending on the food product [28].

#### 2.1.2. Agar

**Figure 3:**
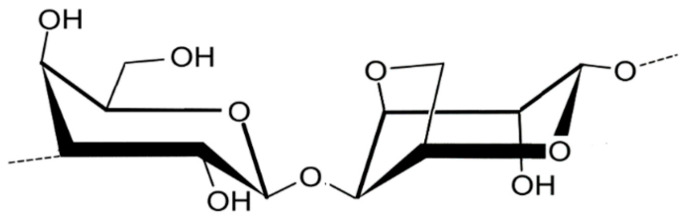
Chemical structure of agar.

Agar (Figure 3) is a polysaccharide characteristic of red seaweed (Rhodophyta) belonging to the orders Gelidiales and Gracilariales.

Agar is a polysaccharide composed of agarose and agaropectin molecules, composed of (1-4)-3,6-anhydro-L-galactose and β9(1-3)-D-galactose residues [31]. To date, there is no evidence that its molecular weight has any significance for food safety, and therefore, it is considered safe regardless of its molecular weight [32].

**Figure 4:**
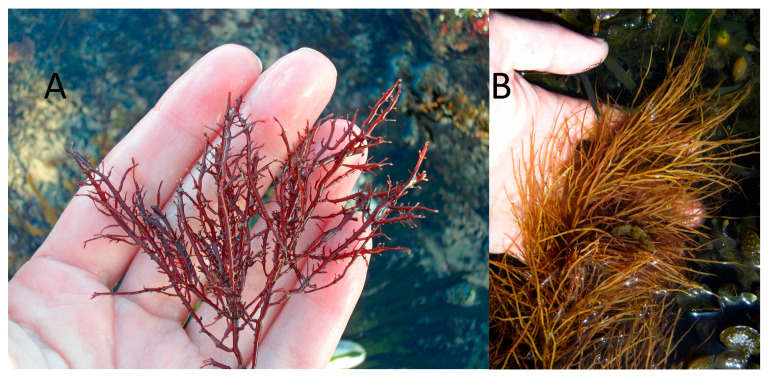
Gelidiales and Gracilariales seaweeds, respectively: (**A**)—*Gelidium corneum*; (**B**)—*Gracilaria gracilis* (Rhodophyta).

The quality of the final product varies greatly between species belonging to these two orders. For example, agar extracted from *Gelidium corneum* (Gelidiales) (Figure 4A) is considered more suitable for pharmaceutical applications due to its high quality and characteristics. It forms strong gels and at higher gelling and melting temperatures. Conversely, agar extracted from *Gracilaria gracilis* (Gracilariales) (Figure 4B) is typically used almost exclusively in the food industry, due to a lower gel strength (compared to *Gelidium*), which can be improved through alkaline treatment, making it suitable and cost-effective for food applications as a gelling, thickening, and stabilizing agent. This agar normally requires an additional step in the industrial extraction process, an alkaline pre-treatment with sodium hydroxide, to enhance the rheological properties of the final product [33,34].

Typically, the industrial extraction method involves heat-treating the seaweed biomass in an aqueous solution (2–4 h at 105–110 °C) followed by immediate filtration of the hot extract (as agar gels very quickly at 50 °C). After the filtration process, the extract gels become a viscous solution due to the amount of agar present. However, the gel itself is typically yellowish or brown in colour due to the degradation of some of its constituents such as proteins and monosaccharides. To achieve this, the freeze–thaw technique is used to obtain a concentrated, clear-coloured agar. This technique allows the agar to be washed with water, thus avoiding the need for an additional pre-treatment step to reduce impurities during extraction. Finally, the resulting agar is dried in an oven with air circulation and then milled for subsequent industrial use [33,35]. Agar is considered safe for human consumption by the regulatory authorities in the United States (FDA) and the European Union (EFSA). Despite the inclusion of agar (E406) in the list of approved food additives (Table 2), its use in food products is regulated and limited. It is estimated that approximately 90% of commercially available agar is intended for the food industry [33].

A study validated by EFSA reveals that at the concentrations of agar tested in rats (dosages of up to 2500 mg of agar/kg of body weight per day), no carcinogenic effects were observed [32]. However, some cases of harmful effects following agar consumption in humans have been reported. For example, the presence of small agar granules (“gastric pouch bezoar”—a mass of undigestible material that accumulates in the small intestine pouch) was reported in women over 64 years of age with a history of obesity and diabetes after consuming agar-based products [36,37]. This case was reported due to the appearance of nausea, vomiting, and dysphagia symptoms in the patient over 72 h after intake of an agar-agar-based dessert and aggravating to postprandial vomiting and unintentional weight loss of 5 kg after 2 weeks, before a CT scan and removal by endoscopy. However, these cases occurred as isolated incidents and were therefore not considered relevant to the assessment of the food safety of agar as a food additive, due to the patients’ clinical histories [32].

#### 2.1.3. Carrageenan

**Figure 5:**
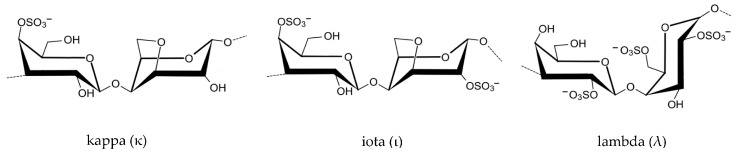
Chemical structure of the different types of commercial carrageenans.

**Figure 6:**
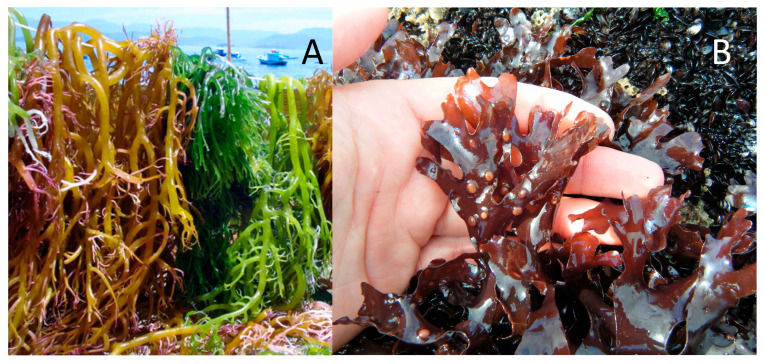
Carrageenophytes seaweeds: (**A**)—*Kappaphycus alvarezii*; (**B**)—*Chondrus crispus* (Rhodophyta).

Carrageenan is extracted from red seaweed of the order Gigartinales. The first historical use of carrageenan was for dietary purposes and occurred in Ireland [38]. Carrageenan is a polysaccharide composed of galactose and 3,6-anhydrogalactose units (Table 3) alternately linked by alternating α-1,3 and β-1,4 glycosidic bonds. A molecular weight greater than 100 kDa is required for safe dietary use [15,39]. Three types of carrageenan (Figure 5) are commonly marketed: kappa-carrageenan (κ) forms rigid gels (Figure 5 with syneresis, commonly extracted from *Kappaphycus alvarezii* (Figure 6A), while iota-carrageenan (ι) (Figure 5 is characterized by producing elastic and soft gels. Finally, lambda-carrageenan (λ) produces viscous solutions (Figure 5 without ever gelling (tetrasporophyte of *Chondrus crispus* (Figure 6B)) [33].

In the carrageenan extraction industry, seaweed pretreatment through a depigmentation step (with organic solvents or sodium hypochlorite) is necessary to obtain a light-coloured final product [38]. The carrageenan extraction step must be carried out in an alkaline (e.g., sodium hydroxide) aqueous solution. Subsequently, carrageenan can be recovered by alcoholic precipitation, drum drying, or precipitation in aqueous potassium chloride with subsequent freezing (as in the case of κ-carrageenan). However, only methanol, ethanol, and isopropanol can be used for carrageenan precipitation and purification [4,40]. To ensure quality and food safety, carrageenan is heat-dried at temperatures above 40 °C in a forced-ventilation drying oven before use [40]. In the European Union, carrageenan, regardless of its type, and carrageenan obtained through *Eucheuma* must, respectively, be labelled as E407 and E407a on food products. The latter is not obtained through an extractive process or a light extraction maintaining the structural backbone, but rather through a process of dissolving other compounds in seaweed [40]. As a rule, carrageenan (E407 and E407a) is used in food products in doses lower than 1 g/L and is authorized by the Joint Committee of the Food and Agriculture Organization and the World Health Organization, except in baby food [41].

**Table 3:** Purity & Specifications for Food-Grade Carrageenan [4,6,8,10,12,40,42].

Parameter	Specification
Description	Yellowish to tan powder; odourless; tasteless; dissolves in hot water to form viscous solutions; gels in presence of certain cations
Identification	Positive for sulphate esters (~15–40% *w*/*w* as SO_4_^2−^) and galactose units; IR spectrum matches carrageenan reference; positive gelation with potassium chloride
Loss on Drying	≤12% (105 °C, 4 h)
Ash Content (as sodium/potassium/calcium salts)	15–40%
pH (1% solution)	8.0–11.0
Viscosity	Defined by grade, typically ≥ 5 mPa·s
Sulphate Content	15–40% (*w*/*w*, as SO_4_^2−^)
Acid-insoluble Matter	≤2%
Lead (Pb)	≤2 mg/kg
Arsenic (As)	≤3 mg/kg
Cadmium (Cd)	≤1 mg/kg
Mercury (Hg)	≤1 mg/kg
Formaldehyde (if used in processing)	≤50 mg/kg
Microbiological Quality	Absence of *Salmonella* in 25 g; absence of *E. coli* in 1 g; total aerobic plate count within GMP limits
Poligeenan Content (low-MW degraded carrageenan)	Not detectable at >5% of total mass; MW distribution profile consistent with undegraded carrageenan (>100 kDa majority)
Functionality	Thickener, stabilizer, gelling agent, water binder, suspension aid

The FDA has determined that the concentration of carrageenan currently used in food products processed in the United States is safe, and thus no upper limit should be established. The FDA only requires that the carrageenan used has a sulphate content between 20 and 40% and that most of the content consists of galactose and anhydrogalactose monomers [43].

However, there is a gap that needs to be addressed: determining the chemical stability of carrageenan (E407 and E407a) after the digestion process [40,44]. This is the opposite of what happens with the other two phycocolloids (agar and alginate) used in the food industry, which are considered safe and stable after digestion, as they are not fragmented by gastric acid in in vitro model systems [45,46,47].

Unlike agar and alginate, carrageenans are sulphated polysaccharides composed of repeating galactose units with distinct amounts and placements of sulphate groups. This sulfation generates a high negative charge, which has a significant impact on their rheological characteristics, modifying solubility, viscosity, and gelling behaviour in the presence of particular cations. For example, κ-carrageenan (one sulphate group per disaccharide) forms strong, brittle gels with potassium ions; ι-carrageenan (two sulphate groups) generates softer, elastic gels with calcium ions; and λ-carrageenan (three sulphate groups) does not for a gel but considerably increase the solution viscosity. The degree of sulfation also contributes to carrageenans’ resistance to enzymatic digestion, allowing them to enter the colon relatively intact. Although food-grade carrageenans are usually considered safe, there has been some worry about their ability to breakdown into low-molecular-weight, highly sulphated fragments (poligeenan), which have been linked to gastrointestinal inflammation in mice. Thus, sulfation is an important structural property that not only dictates the functional capabilities of carrageenans in culinary applications but may also explain some of their controversial health consequences [3,6,9,10,11,12,17,39,40,42,44,48,49,50,51].

In non-standard in vitro gastric digestion assays, carrageenan has yielded mixed results, with some studies demonstrating complete or partial hydrolysis of the molecule [11,49,52]. However, in other studies, carrageenan is not hydrolysed after handling behaving similarly to other phycocolloids and being considered safe for human consumption [15,50,51,53]. In this case, the degradation or non-degradation of carrageenan is an essential question to determine the level of risk to human health, since the degraded molecule has an inflammatory effect and is thus considered harmful to human health [15,49]. Additionally, there is a need for a standard human simulated digestion model to give safe information and avoid variations in results due to methodological differences

## 3. Carrageenan in the Human Health

Although phycocolloids (commercial seaweed polysaccharides) are considered safe for human consumption, few studies demonstrate this safety, especially when it comes to alginate and agar. In the case of carrageenan, there is considerable debate and quite divergent results [10,11,12,17,42,44,53,54]. These phycocolloids (and not just carrageenan) must be analysed and approved for human consumption due to food safety concerns [4,24,32,40]. However, the lack of chemical characterization of phycocolloids hinders a correct safety assessment, as the methods used, from extraction to analysis of their bioactivity, are diverse and incomparable.

It is known, however, that these polymers should not have low molecular weight, as this could cause cellular damage, thereby interfering with the normal functioning of human cells [15]. The most commonly described effects in the literature are the induction of intestinal tumours, gastric ulcers, and inflammation [11,55]. In several gastric digestion studies, carrageenan is degraded during digestion, leading to its consideration as a potential public health hazard [11,17,48,56]. Post-digestion carrageenan (with no defined molecular weight) induces inflammation in several intestinal cell lines (NCM460, HT-29 and HCT-8) and may be absorbed into blood plasma, inducing oxidative stress and a subsequent pro-inflammatory effect [32]. On the other hand, there are studies on gastric digestion of carrageenan that demonstrate no degradation of the compound after gastric digestion (without known molecular weight or physicochemical profile). Therefore, digested carrageenan does not interfere with the digestive system, being considered a safe dietary fiber with no impact on human cells, but only on the intestinal microbiota [12,39,50,51,53]. The disparities found in the literature on carrageenan degradation and its biological effects can be attributed to variances in the analytical methods, experimental conditions, carrageenan type and purity. Food-grade carrageenans are high-molecular-weight polysaccharides. Degraded carrageenan (poligeenan) with a molecular weight < 20 kDa has been linked to gastrointestinal inflammation, ulceration, and tumour promotion. Some research found that negative effects may have employed or manufactured low-molecular-weight fragments under harsh acidic or heat conditions, but others proved the stability of food-grade carrageenan throughout physiologically appropriate stomach digestion. Furthermore, In vitro investigations on intestinal cell lines frequently use high dosages or indeterminate molecular weight fractions, which may not accurately represent in vivo exposures. When carrageenans decompose in digestion tract, they produce low-molecular-weight galactose- and anhydrogalactose-based oligosaccharides and sulphate esters free group, frequently with sulphate substitutions, which are more bioactive and may induce oxidative stress and inflammation. Thus, the variety in study results is most likely due to changes in the structural integrity of carrageenan throughout digestion, with high-molecular-weight food-grade carrageenan typically deemed safe and degraded, low-molecular-weight forms causing toxicological concern [4,9,10,11,12,14,52,57].

However, few studies present data on the chemical characterization (specifically, molecular weight and molecular conformation) of carrageenan before and after digestion, which complicates a critical analysis [11,15,39,48,52,56]. This scarcity of data on the chemical characterization of carrageenan after digestion makes it impossible to properly evaluate changes in its molecular weight and structure. Thus, this molecular weight and critical data is essential to understand the reactions on the chemical structure of phycocolloids during digestion models. Moreover, no detailed studies on this topic were found for carrageenan, agar or alginate. A perfect example of this issue is poligeenan, a degraded ι-carrageenan with a molecular weight between 10–20 kDa, which is not authorized for dietary use because it is associated with adverse effects and is used as a pro-inflammatory agent in vitro and in vivo assays [15,39]. Another issue raised after analysing the literature is that there is no standard methodology for carrageenan digestion, as each group has its own methodology for both digestion and bioactivity analysis. Therefore, comparative analysis will always have an associated error, as referenced in the review by McKim et al. [12]. This review [12] also highlights that there are digestion methods in use that have been compared to human gastric digestion but are not actually similar. For example, digestion time, acid concentration, and temperature are very different from those found in normal human gastric digestion.

However, there is also evidence that undegraded carrageenan has beneficial bioactivities for the body, such as the modification of intestinal microbiota [58,59,60]. Carrageenan supplementation in animal models has been linked to an increase in short-chain fatty acid (SCFA)-producing bacteria, such as *Bacteroides* and *Lactobacillus*, as well as a decrease in potentially harmful taxa [61]. These changes are associated with increased synthesis of acetate, propionate, and butyrate, which promote gut barrier integrity and immunological homeostasis. Although, the carrageenan high intake can enhance the consumption of nutrients from intestinal mucus by gut bacteria, exceeding SCFA production. This phenomenon has been linked to the disruption of intestinal integrity [61,62,63]. Overall, while the present data comes from animal research, carrageenans’ potential to modify gut microbial populations implies they may operate as fermentable dietary fibers with prebiotic-like qualities. And the type of carrageenan can also have distinct behaviour from lambda to kappa carrageenan due to their chemical structure change [4,10,11,39,40,62,64].

In addition to being considered dietary fiber, phycocolloids possess innate properties that are very important for intestinal health (high water retention capacity and viscosity), especially those that promote satiety and weight loss. Additionally, they delay the end of gastric digestion, thus promoting glycaemic control (i.e., reducing the incidence of diabetes). In the intestinal tract, all seaweed-derived phycocolloids increase intestinal transit, maintaining a regular stool volume. Thus, phycocolloids promote positive impacts on the gastrointestinal system, resulting in improved cardiometabolic, immunological, and mental status [65,66,67]. Although, there are no published results in the simulated digestion of seaweed polysaccharides using a consensual method using simulated human digestion.

## 4. Seaweed Polysaccharides and Gastrointestinal Interaction

**Figure 7:**
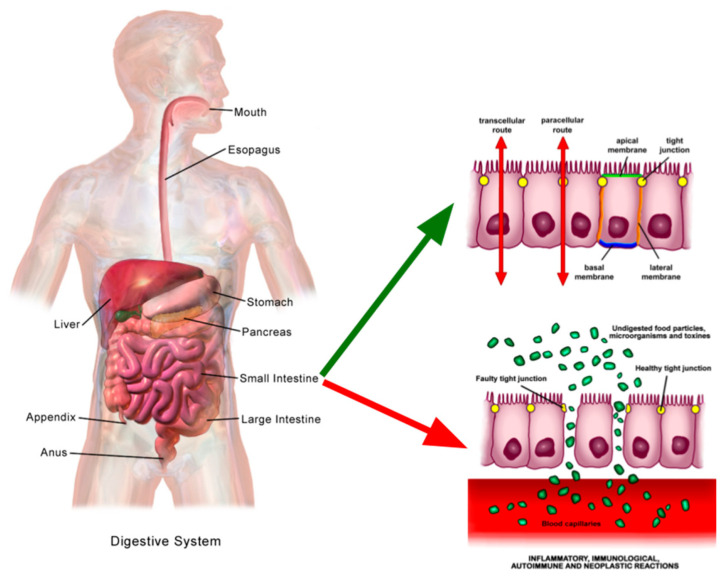
Human digestion apparatus, in the two main “entries” of nutrients to the human blood stream, buccal and intestinal (after digestion) (Original work of BallenaBlanca and BruceBlaus gathered from Wikimedia, remixed by the authors based in Creative Commons Attribution-Share Alike 4.0 International). Available online: https://commons.wikimedia.org/wiki/File:Adult_Digestive_System.png (accessed on 23 September 2025). https://commons.wikimedia.org/wiki/File:Increased_intestinal_permeability.png (accessed on 23 September 2025). https://commons.wikimedia.org/wiki/File:Selective_permeability_routes_in_epithelium.png (accessed on 23 September 2025).

Human digestion is an intricate procedure that requires the coordinated activity of digestive enzymes on food substances. A variety of factors impact the digestion of food, such as gastric and intestinal pH, digestive enzyme activity, endogenous secretions, and motility. Digestion begins with a brief chewing stage in the mouth, which is important for the whole digestive process, notably the stomach emptying rate. Peristaltic motions convey the food bolus formed by mechanical and enzymatic breakdown in the mouth via the oesophagus to the stomach. When the bolus enters the proximal section of the stomach, it is combined with gastric juice, which is largely made up of hydrochloric acid (HCl), salts (mostly calcium and sodium chloride), pepsin, and lipases, which are responsible for protein and fat digestion, respectively, as well as mucus, which protects the stomach mucosa. The stomach finishes at the pylorus, which pumps minute particles (chyme) to the duodenum, while larger particles remain in the stomach for further processing. When the chyme enters the duodenum, its acidic pH is neutralized by sodium carbonate until it reaches a pH suitable for the activity of pancreatic (proteases, amylases, and lipases) and intestinal enzymes, which are responsible for the subsequent digestion of molecules in the chyme. The liver produces bile, which aids in lipid digestion by emulsifying dietary lipids into tiny droplets that promote lipase action. Once digested, released nutrients are accessible for absorption by villus enterocytes via various transport processes, whereas unabsorbed material goes to the large intestine. In the colon, water and electrolytes are absorbed, bile salts are reabsorbed, and colonic bacteria degrade non-digested polysaccharides and proteins, producing new breakdown products. Finally, near the end of the large intestine, faeces are formed, stored, and eliminated [68,69,70]. In vitro digestion models frequently fail to adequately replicate these complicated processes, as discussed above. This information is highly important, because this needs to be accounted when the researchers are developing human simulated gastric digestion methods, where some of the methods used to degrade carrageenans, are very different from the above established [10,11,12]. Although, what we eat can be benefit or prejudicial to the intestinal mucosa layer and develop human health problems (Figure 7), so one of the keystone to be observed and answered is if the seaweed polysaccharides promote a healthy intestinal mucosa or can be a key factor for intestinal mucosa degradation, this can be observed using membranes system or using pig intestine cleaned, which are used in traditional sausages [13].

### 4.1. Stability and Hydrolysis in the Digestive Tract

The human gastrointestinal tract faces a variety of physical and chemical challenges, including pH variations, mechanical mixing, enzymatic hydrolysis, and bile salt interaction [57]. Most seaweed polysaccharides withstand enzymatic hydrolysis in the upper gastrointestinal tract and are subsequently fermented in the colon [10]. This resilience is a key factor in their utility as dietary fibres, which promotes gut health and helps to modulate composition of gut microbiota [3,71]. Although, their chemical modification in stomach can alter their functional and safety consequences in food applications [4].

Unlike simple carbohydrates, the seaweed polysaccharides are largely resistant to enzymatic breakdown by human digestive enzymes due to their unique glycosidic linkages and the presence of sulphate groups or uronic acids potential partial hydrolysis can occur in the acidic environment of the stomach or through the action of microbial enzymes in the colon, this aspect is inconclusive in the analysed literature. Subsequently, resident bacteria in the large intestine can ferment some of these polysaccharides, leading to the production of short-chain fatty acids (SCFAs), such as acetate, propionate, and butyrate, which are known to promote intestinal health [61]. The extent and pattern of this fermentation are determined by the polysaccharide structure and the individual’s microbiome [67,71,72,73]. So, there are variances in the amount and composition of SCFAs produced during intestinal fermentation. The in vitro and in vivo results suggest that overall SCFA synthesis is higher for alginate and its derivatives than for undegraded carrageenan, with agar falling somewhere in the middle, although frequently poor unless depolymerized. Acetate is the predominant SCFA in most situations for all three polymers, however butyrate is often higher in alginate oligosaccharides and agarose/agar-derived oligosaccharides than in carrageenan studies. Lower molecular weight polymers or oligosaccharide forms are more fermentable than high molecular weight forms. The overall composition of the microbiota also has a significant impact on the kind of SCFA generated [74,75,76,77,78]. Although, the most data on this subject comes from in vivo experiments, in vitro models often fail to accurately simulate the complex conditions of human digestion, particularly those required to study the polysaccharide structure which is present at intestinal phase.

Therefore, understanding the polysaccharide chemical degradation profile and stability of these compounds is crucial for assessing their safety, especially regarding the potential formation of bioactive low molecular components and their interactions with the intestinal mucosal barrier. Additionally, the persistence of high-molecular-weight polysaccharides in the gastrointestinal tract indicates limited systemic absorption, thereby reinforcing their classification as safe dietary fibres with a low toxicological risk.

### 4.2. Gaps in Current Digestive Assays

Current in vitro digestive assays lack standardization due to a variety of factors, including differences in acid concentrations, incubation times and temperatures, enzyme composition, mechanical agitation, and sample preparation methods. These discrepancies often lead to conflicting findings. Furthermore, these assays frequently fail to account for non-enzymatic degradation pathways, such as oxidation or interaction with food components [70,79].

Despite the growing interest in seaweed polysaccharides for food and health applications, current digestive assay models have significant limitations in assessing their gastrointestinal behaviour. Most typical established in vitro digestion models, such as the standardized INFOGEST technique, are primarily designed for the evaluation of proteins, lipids, and simple carbohydrates, not contemplating the marine polysaccharides [7].

The most notable limitation of current digestion models is their incomplete integration with data analysis. These models often lack a comprehensive physicochemical parameterization (e.g., pH, electrical conductivity and dissolved solids) and chemical analysis of the digestate products, which goes beyond the study of microbial fermentation in the human colon. As many seaweed polysaccharides are resistant to enzymatic hydrolysis in the upper gastrointestinal system, their principal transformation occurs in the colon via microbial fermentation. However, current models frequently exclude or oversimplify this stage, failing to account for critical factors such as interindividual variation in gut microbiota composition, enzyme activity, and fermentation outcomes, including short-chain fatty acid synthesis [7,70].

Furthermore, the simple digestive assays (using only HCl but varying its concentration) usually neglect aspects such as polysaccharide molecular weight, sulfation patterns, and ionic crosslinking, all of which significantly influence their digestion and bioavailability [80]. Therefore, the absence of standardized methods for evaluating the degradation and transformation of high-molecular-weight and chemically complex polysaccharides, such as carrageenans, is a direct consequence of this methodological gap, leading to conflicting findings among investigators.

Another significant challenge lies in determining whether bioactive or immunomodulatory fragments may develop after digestion. Current assays and studies are frequently not designed to detect low-abundance hydrolysis products or to properly assess their biological significance. This methodological limitation, in turn, constrains the comprehensive evaluation of the safety and functional implications of seaweed polysaccharides in the digestive environment.

To address these gaps, the adoption of more physiologically relevant and dynamic models is essential, including enhanced colonic fermentation systems, gut-on-a-chip platforms, and standardized microbiota simulations. These advancements would enable more accurate safety evaluations, and a deeper understanding of how these complex dietary fibers interact with human physiology [7].

### 4.3. Digestion and Hydrolysis: Understanding Molecular Stability

Due to their complex structure and β-linkages, seaweed polysaccharides exhibit resistance to salivary and pancreatic enzymes. Once in the colon, these polysaccharides are fermented by the gut microbiota leading to the production of SCFAs. The SCFAs have been associated with enhanced colonocyte health and the modulation of inflammatory responses. Conversely, lower concentrations of SCFAs are more likely to lead to the disintegration of the intestinal mucosa [14,81,82].

In contrast to starches and common plant fibres, several seaweed-derived polysaccharides, exhibit notable stability under the acidic and enzymatic conditions, this resistance is attributed to their unique structural features, including β-linkages, sulfation, and gel-forming properties, which prevent degradation by amylase and protease [14].

Hydrolytic stability is a very important characteristic in food safety research because it determines whether polysaccharides will yield potentially bioactive, immunogenic, or toxic fragments after digestion. For example, low-molecular-weight carrageenan fragments generated under specific hydrolytic conditions have prompted safety concerns due to potential pro-inflammatory effects. Therefore, a thorough understanding of the specific conditions that induce such degradation, including pH, temperature, ionic strength, and microbial enzyme activity, is essential for accurately assessing their safety in food applications. This knowledge also helps to fill gaps in risk assessment processes by establishing a clear correlation between structural characteristics, digestive fate, and physiological effects [83,84].

## 5. A Rational Framework for Future Studies

Many variables impact digestion processes, including gastric emptying, intestinal transit, production of digestive fluids and mucus, and motility. Specifically, low pH and temperature, high osmolality, viscosity, fibre content, and energy density (caloric content) have all been shown to delay stomach emptying, whereas a larger meal volume tends to accelerate this process. Furthermore, the particle size and degree of hydrolysis of meal ingredients can also exert a significant influence [70,85,86,87].

### 5.1. Models of Simulated Digestion

Animal models frequently involve invasive procedures, such as the surgical placement of catheters into digestive organs to access gastrointestinal content, an often culminate in the death of the animals. Consequently, in vivo investigations are associated with considerable ethical constraints, in addition to being expensive and time-consuming. To address these limitations, various vitro gastric and small intestine models have been developed and refined in recent years [70].

Static models are the most widely used digestive systems due to their cost-effectiveness, simplicity of operation, and minimal equipment requirements. These models consist of a series of bioreactors that simulate the physicochemical and enzymatic conditions of each digestive compartment. The most basic and common in vitro stomach digestion model is a water bath incubation model, which may additionally incorporate digestive enzymes (amylases, proteases, and lipases), mucins, and salts, depending on the food product being studied and the specific gastrointestinal region been simulated. In a typical protocol, samples are combined with a simulated digestion solution (at the appropriate pH and enzyme concentration) and incubated at 37 °C for 2–3 h. Some models may include agitation to simulate the peristaltic motions found in the gastrointestinal system, as well as a pH meter to monitor the pH of the digest at regular intervals during the digestion process. A significant drawback of these methods is the lack of standardized experimental conditions (e.g., pH and length of the separate phases, amount of digestive enzymes and bile salts), which makes the comparison of results across different studies extremely challenging [70,88,89,90].

Dynamic systems can be either mono-compartmental (simulating only one compartment of the gastrointestinal tract) or multi-compartmental. These models can execute continuous pH changes and sequential secretion of gastric or pancreatic juices, as well as gastric emptying or digestive product removal. As a result, dynamic models are regarded as the ideal systems for replicating physiological circumstances due to their ability to imitate both mechanical and enzymatic processes that occur during gastrointestinal digestion. These models are quite complex, and the set-up and maintenance expenses are extremely expensive. Furthermore, to correctly imitate in vivo digesting conditions, the primary parameters must be programmed using in vivo data [70,91,92].

### 5.2. Limitations of Current In Vitro Digestion Models

In vitro digestion models have become critical tools for studying the behaviour of food substances in the human gastrointestinal system. However, when applied to complex seaweed polysaccharides, these models show substantial limitations, which may jeopardize the accuracy of safety and functionality assessments [93,94].

Most modern in vitro models, such as the widely used INFOGEST protocol, are primarily designed for the digestion of macronutrients including proteins, lipids, and easily hydrolysed carbohydrates [95]. These models frequently assume enzymatic accessibility and solubility, which are not applicable to high-molecular-weight, structurally varied marine polysaccharides. This challenge is further highlighted by the scarcity of published data on the digestion of whole seaweed or their individual components using these models [96,97].

A significant limitation is the absence of real-time monitoring of molecular degradation. Current models mostly rely on endpoint assessments, which may overlook temporary. This limits our ability to identify toxic or bioactive fragments that may affect mucosal integrity, immune response, or microbial ecology [70].

Furthermore, most in vitro digesting methods do not include mechanical modelling [98]. These interactions have a considerable impact on digestibility, bio-functionality, and safety.

To address these constraints, additional physiologically appropriate models—such as dynamic gastrointestinal simulators, microfluidic gut-on-a-chip platforms, and colonic bioreactors—should be included in seaweed polysaccharide research. These sophisticated technologies better simulate the complex environment of the human gut, allowing for a more precise assessment of the digestion, hydrolysis, and possible health effects of these intriguing but structurally complicated dietary constituents [7].

### 5.3. Need for Standardized Digestion Assays

As seaweed-derived polysaccharides rise in the food and nutraceutical industries, and inherently their possible human dangers there is a pressing need for standardized, reliable digestion tests that can reliably assess their behaviour and safety in the human gastrointestinal system. While the INFOGEST static in vitro digestion model is a widely accepted procedure for modelling human digestion, its current framework has limitations when applied to complex marine polysaccharides [93].

The INFOGEST model was created primarily to investigate the digestion of conventional macronutrients, including proteins, lipids, and simple carbohydrates, under controlled conditions that simulate the oral, gastric, and small intestine phases. Furthermore, INFOGEST assumes uniform enzymatic and pH conditions, whereas the degradation and solubility of seaweed polysaccharides can be highly sensitive to ionic strength, pH variability, and the presence of bile salts, factors that are especially important given the gel-forming and ion-binding nature of many marine polysaccharides [95,99,100].

Standardizing digestive tests for seaweed polysaccharides as other food components is critical not only for regulatory safety evaluations, but also for realizing their full potential as functional dietary components and prebiotics [64,77].

### 5.4. Simulating Physical and Chemical Reactions

Standardized models should account for crucial factors such as stomach motility, peristalsis, mucosal interaction, and the existence of reactive oxygen/nitrogen species, which are frequently overlooked in current digestion models [96]. Given the complexity of marine polysaccharides, which have high molecular weight, various glycosidic connections, and distinctive modifications such as sulfation, realistic and integrative modelling methodologies are required to correctly reproduce their behaviour [7,101]. These simulations serve to recreate circumstances seen in the human gut, such as fluctuating pH levels, digestive enzyme exposure, bile salt interaction, and microbial fermentation [7,101].

Polysaccharides undergo physical processes such as viscosity alterations when they move from highly organized macromolecules to partially degraded, low-viscosity fragments. These modifications can influence transit duration, nutrition encapsulation, and interactions with gut mucosa. Swelling, gel formation, or breakdown can also influence satiety, medication delivery, or toxin binding, especially in the case of alginates and carrageenans [102,103].

Although most seaweed polysaccharides are resistant to human digestive enzymes, chemical processes such as pH-dependent ion dissociation (e.g., calcium or magnesium from alginates) or sulphate release (from fucoidans or carrageenans) may still occur. These processes may influence the polysaccharide’s functional groups and how they interact with other nutrients or gastrointestinal receptors [104].

### 5.5. Molecular Integrity and Degradation Tracking

Monitoring the molecular integrity and breakdown of food components throughout digestion is critical for determining their functional potential, safety, and bioavailability. As a result, improved and quick characterization approaches are increasingly needed to precisely track their progression through the gastrointestinal system.

Importantly, the use of dialysis-based systems—which are frequently used to replicate the intestinal barrier—provides vital insights on how much of a digestate might penetrate the epithelial layer and potentially enter systemic circulation. On such tests, digestion mixes are deposited on dialysis membranes with molecular weight cut-offs that simulate the size-selective permeability of the small intestinal barrier. This setup distinguishes between high-molecular-weight polysaccharides, which normally remain in the lumen and are subsequently fermented in the colon, and low-molecular-weight fragments or di- or saccharides (e.g., sulphated galactose, radical of sulphated esters, uronic acids, mannitol) which can pass through and have systemic effects [16,77,105,106,107,108].

By combining these dialysis approaches with rapid analytical profiling, researchers may determine not only if molecular degradation occurs, but also the biological significance of the resulting metabolites. While most seaweed polysaccharides are considered non-absorbable dietary fibers, partial breakdowns under digestive or microbial conditions can yield small bioactive fragments with immunomodulatory or pro-inflammatory properties. Therefore, the identification and quantification of these fragments is essential for a full risk assessment.

Despite recent advancements, there is still a lack of defined methods for combining dialysis-based tests with digesting models like INFOGEST. Harmonizing these approaches with high-resolution analytical platforms would significantly enhance the reliability of degradation tracking and intestinal transit investigations. Finally, the development and implementation of rapid, sensitive, and physiologically relevant characterization methods is critical for furthering our understanding of seaweed polysaccharide digestion and assuring their safe and effective application in food systems [7].

Understanding the structural integrity and breakdown behaviour of seaweed polysaccharides during digestion is critical for determining their safety, functionality, and bioavailability. These complex macromolecules frequently resist hydrolysis in the upper gastrointestinal system, but may be partially degraded in the colon, where microbial fermentation produces a variety of oligosaccharides and metabolites. To capture these alterations in a physiologically relevant way, a combination of modern analytical methods and barrier simulation models is required [7].

#### 5.5.1. FTIR-ATR

Fourier-Transform Infrared Spectroscopy with Attenuated Total Reflectance is an important and increasingly utilized method. FTIR-ATR enables quick and non-destructive investigation of chemical changes in polysaccharide structures, including the identification of sulfation patterns, uronic acid residues, and glycosidic connections. During digestion, FTIR-ATR can detect spectrum changes that indicate molecule breakdown or the synthesis of new functional groups. This is especially important for assessing the stability of sulphated polysaccharides such as carrageenans and fucoidans, since sulphate loss or bond breakage might suggest major molecular changes [7].

When FTIR-ATR is used in combination with physicochemical factors like viscosity, pH, and electrical conductivity, it provides a more complete picture of the digestive process. Viscosity assays can reveal variations in molecular weight and gel-forming capability, which are essential for understanding how polysaccharides function as dietary fibers in the gut. A decrease in viscosity during simulated digestion indicates polymer breakdown or depolymerization, which may be associated with FTIR-detected bond breaking [109].

#### 5.5.2. NMR and Advanced Analytical Techniques

Advanced analytical techniques, such as nuclear magnetic resonance (NMR) spectroscopy, mass spectrometry, and chromatographic methods, can help characterize digestate products by identifying specific oligosaccharides or bioactive fragments that may influence gut physiology or systemic immune responses. Combining these methods with dialysis-based absorption tests provides a complete framework for investigating the fate, bioavailability, and possible health impacts of seaweed polysaccharides.

NMR spectroscopy can reveal comprehensive structural information on monosaccharide composition, glycosidic linkages, and sulfation or uronic acid substitution patterns. Its main strength is clear structural elucidation, which does not need derivatization. However, NMR is often a costly and highly specialized technique that requires large sample amounts and high purity, and its sensitivity is lower than MS-based approaches [110,111].

Mass spectrometry, particularly when combined with chromatographic separation (e.g., LC-MS or GC-MS), offers high sensitivity and can identify low-abundance metabolites and small degradation products. MS is excellent at profiling molecular masses and fragmentation patterns, making it ideal for detecting oligosaccharides and their sulfation states. However, its limitations include the necessity for meticulous sample preparation, matrix effects, and occasionally difficulty differentiating isomeric structures without additional procedures [112,113,114].

Polysaccharides and oligosaccharides are commonly separated using chromatographic techniques (such as HPLC, UHPLC, and size-exclusion chromatography) based on size, charge, or content [115,116]. They give quantitative data on molecular weight distribution and degradation rates and can be used with MS or NMR for structural investigation. However, chromatography alone frequently lacks the structural resolution required to determine exact linkage patterns or substitution sites, making it best suited as a supplementary method.

These techniques take more time than the FTIR-ATR; however, they can complement the information more completely for the digestion assay.

#### 5.5.3. Electrochemical Analysis

pH monitoring is also necessary since seaweed polysaccharides can buffer stomach and intestinal pH, modify the local environment and influence enzyme function. As a result, pH changes can affect their solubility, structure, and capacity for hydrolysis [117]. A reduction in pH, for example, may increase acid-catalysed hydrolysis of some polysaccharides, as seen by contemporaneous FTIR and viscosity changes.

Electrical conductivity is an indirect indicator of ionic species produced during digestion, such as sulphate, uronic acids, or monovalent ions resulting from ion exchange interactions. An increase in conductivity may indicate molecular dissociation or breakdown, particularly in ion-rich polysaccharides such as alginates and fucoidans [4,118]. When used with FTIR-ATR, conductivity measurements can help determine when and how ionically bound functional groups are released.

#### 5.5.4. Final Remarks

To determine the bioavailability of degradation products, dialysis-based methods that simulate the intestinal barrier are used [119]. These models help researchers to determine which molecular fragments are tiny enough to pass through the gut lining. When combined with FTIR, viscosity, pH, and conductivity measurements, dialysis gives vital information on whether complete polysaccharides stay in the gut lumen or if smaller bioactive fragments enter systemic circulation. Since some of the equipment is used as food quality control by the phycocolloid industry, there is the possibility to develop chemometrics systems using official quality control data. If the standard method is simple and feasible, the industry may be open to support the larger studies promoting human welfare.

However, no analytical technique is perfect (Table 4), there is a need of complementarity with other data acquisition, such as pH, redox potential and conductivity measurements. This multi-parametric technique, which combines FTIR-ATR spectroscopy, viscosity profiling, pH monitoring, electrical conductivity, and dialysis simulation, provides a robust, real-time assessment of seaweed polysaccharide integrity and transformation during digestion. It not only improves our mechanistic understanding of digestion but also aids in risk assessments and the development of functionally optimal marine-based food additives.

### 5.6. Microbiome Interaction and Metabolite Profiling

The interaction of seaweed polysaccharides and the gut microbiota is crucial for their impact on human health. Understanding this interaction by microbiota research and metabolite profiling is critical for determining the health benefits and safety implications of these chemicals [70].

Their fermentation is frequently restricted to select microbial taxa with the genetic ability to handle marine glycans. These include the Bacteroidetes phyla, specifically *Bacteroides* and *Prevotella* genera, which possess polysaccharide utilization loci (PULs) capable of detecting and degrading marine-derived polysaccharides [120].

Seaweed polysaccharides may be transformed into SCFAs; however, the type and amount of SCFAs in humans are mostly unknown [14,64]. Metabolite profiling, which is commonly performed using gas chromatography-mass spectrometry (GC-MS), nuclear magnetic resonance (NMR), or liquid chromatography-mass spectrometry (LC-MS), enables the identification of fermentation products and bioactive intermediates produced during polysaccharide degradation. These include SCFAs, lactate, phenolic derivatives, and potentially immunomodulatory oligosaccharides. Monitoring these molecules offers information on the functional effects of microbiome-polysaccharide interactions.

Moreover, some bacteria potentially metabolize highly sulphated seaweed polysaccharides. Sulfatase-producing bacteria remove sulphate groups, resulting in free sulphate that sulphate-reducing bacteria convert to hydrogen sulphide (H_2_S). Elevated quantities of H_2_S can harm colon epithelial cells, causing inflammation and genetic damage [121,122,123]. This highlights a critical method for assessing the health risk of these polysaccharides.

Emerging technologies for simulating and studying these interactions include in vitro colon fermentation models, faecal batch cultures, and bioreactor systems. When combined with high-throughput sequencing and metagenomics, these systems can show how unique seaweed polysaccharides alter microbial populations and influence metabolic pathways throughout time.

## 6. Recommendation

Now, several studies use in vitro simulated digestion models to evaluate the bio-accessibility of dietary constituents. Where the variations on the digestive model can make the studies non-comparable, thus it is necessary to establish one standard method. The most frequently examined by INFOGEST foods are dairy products, egg products, meat and seafood products, emulsified foods, fruits and vegetables, and cereal products [96].

Polysaccharides can also affect nutrition, causing reduced diffusion and mass transfer, impeding mixing of digestive components, blocking enzyme active sites, causing conformational changes, and the development of aggregates and surface bonds that immobilize substrates. The degree of digestion inhibition is determined by factors such as polysaccharide content, viscosity, and molecular structure, as well as substrate qualities like molecular weight and conformation. The varied composition of food also influences the amount of hydrolysis and nutrient absorption, since some dietary items might block particular enzymes. Beyond the food matrix, many environmental parameters can further influence digestibility, affecting material behaviour and chemical reactions [84].

Moreover, structure/activity relationships of algal polysaccharides, particularly their glycosidic linkages and sulphate positioning, play a decisive role in determining their biological effects even after digestion. These structural features influence their capacity to modulate gut microbiota and stimulate short-chain fatty acid production, as well as their antioxidant potential and their ability to inhibit key signalling pathways such as mTOR and JAK–STAT3, which are relevant in chronic disease contexts [2].

## 7. Conclusions and Future Perspectives

Seaweed polysaccharides are a promising yet understudied class of dietary components with potential use in digestive health and cancer prevention. A reasonable, consistent approach to digesting research is critical for determining their genuine bioactivity and safety. By improving analytical tools and simulation models, we can bridge the gap between food chemistry and gastrointestinal studies, thereby leading to better public health initiatives.

Cancers of the human digestive system, including the oesophagus, stomach, liver, pancreas, and colon, pose considerable worldwide health risks. They are frequently diagnosed at an advanced stage, resulting in a high fatality rate. Emerging data points to a substantial link between food, digestion, and the development of gastrointestinal diseases, including cancer. However, our understanding of how dietary components interact with the digestive tract remains limited.

This uncertainty about actual or hypothetical negative effects of carrageenans can directly impact public health, disease management and lifestyles. The major barrier to understanding the complete safety profile of these prevalent food additives is the lack of data on the stability of various carrageenan, alginates and agar types throughout food processing and their interactions with dietary constituents in the gastrointestinal tract. Future studies should focus on filling these information gaps regarding the effects of dosage and chronic exposure, as well as the interactions between phycocolloids and other food additives. Furthermore, it is recommended to investigate the intimate interactions between food-grade polysaccharides of varying MW, disaccharide content, and conformation in patients with poor health condition. Studies using other food additives like to carrageenans are also warranted.

As interest in seaweed polysaccharides rises in nutrition, food safety, and medicinal research, there is a critical need to expand and update the scientific frameworks used to assess their digestion, bioactivity, and health implications.

Currently, there is substantial diversity in digestion model design, enzyme doses, residence times, and physicochemical factors, resulting in inconsistent or non-reproducible results between studies. The use of internationally accepted criteria, such as those recommended by the INFOGEST network. Transcriptomics, proteomics, and metabolomics can provide valuable insights into host and microbial responses, including changes in gene expression, metabolic fluxes, and immunological regulation. When combined with established tests such as FTIR-ATR, viscosity, and SCFA profiling, omics technologies can bridge the gap between molecular structure and physiological function.

Long-term human cohort studies are required to evaluate the real-world health effects and safety of seaweed polysaccharides-based products. Such studies should monitor food consumption patterns, gut microbiota changes, indicators of gut barrier function, hepatic and pancreatic alterations, and inflammation throughout time, especially in populations at high risk for gastrointestinal illnesses including cardiovascular diseases, colorectal cancer, or metabolic disorders. Emphasis should be placed on inter-individual diversity in microbiota composition, which might alter polysaccharide fermentation results and host responses. If possible, create and develop a reactor to simulate this type of human function in a humanoid reactor system to protect humans from possible nefarious problems. Applying the advances in artificial intelligence (AI), chemometrics and computer modelling provide transformative tools for predicting digestion on a systemic level. Machine learning algorithms may be trained on vast datasets, which include chemical structure, enzyme kinetics, microbial taxonomy, and clinical results, to simulate digestive dynamics, fermentation profiles, and potential health consequences. These methods may also be used to discover crucial structure–function relationships, optimize polysaccharide formulations, and lead the development of specialized dietary therapies for specific groups.

To fully realize the potential of seaweed polysaccharides in improving gastrointestinal health and assuring food safety, a multidisciplinary approach is required, combining standardized digestion methods, molecular tools, clinical data, and computational insights. These solutions, which are developed via worldwide collaboration, will allow for the creation of evidence-based guidelines, safer food uses, and innovative treatment methods based on marine bioactives.

## Figures and Tables

**Table 1:** Purity & Specifications for Food-Grade Alginate [4,24,27,28].

Parameter	Specification
Description	White to yellowish-brown powder; odourless; tasteless; soluble in water forming viscous colloidal solution
Identification	Positive for uronic acids (mannuronic & guluronic) via chemical test or IR spectrum matching standard alginate
Loss on Drying	≤15% (105 °C, 4 h)
Ash Content (as sodium salt)	18–27%
pH (1% aqueous solution)	3.5–10.0
Insoluble Matter	≤2%
Lead (Pb)	≤2 mg/kg
Arsenic (As)	≤3 mg/kg
Cadmium (Cd)	≤1 mg/kg
Mercury (Hg)	≤1 mg/kg
Formaldehyde (if used in extraction)	≤50 mg/kg
Microbiological Quality	Absence of *Salmonella* spp. in 25 g; absence of *E. coli* in 1 g; total aerobic count within GMP limits
Heavy Metals (as Pb)	≤0.001%
Functionality	Thickener, gelling agent, stabilizer, emulsifier

**Table 2:** Purity & Specifications for Food-Grade Agar [4,8,32].

Parameter	Typical/Recommended Specification
Description	White to pale-yellow translucent flakes or powder; odourless; forms a firm, clear gel in hot water
Identification	Positive for 3,6-anhydro-L-galactose and D-galactose by specific chemical tests or FTIR; melting/setting point profile consistent with agar
Loss on Drying	≤12% (105 °C, 4 h)
Ash Content	Typical: 1–8% (depends on purification; lower for agarose)
pH (1% solution)	~5.0–7.0
Insoluble Matter	≤2%
Gel Strength (Bloom or g/cm^2^ equivalent)	Typical: 400–1200 g/cm^2^ (many food grades ≥ 600 g/cm^2^)
Viscosity (1–2% solution, specified temp)	Specified by grade (e.g., medium/high gel)
Lead (Pb)	≤2 mg/kg (typical limit)
Arsenic (As)	≤3 mg/kg (typical limit)
Cadmium (Cd)	≤1 mg/kg (typical limit)
Mercury (Hg)	≤1 mg/kg (typical limit)
Microbiological Quality	*Salmonella* absent in 25 g; *E. coli* absent in 1 g; total plate count within GMP limits
Residual Solvents/Chemicals	Not detected or below method LOQ (e.g., if solvents were used in processing)
Adulterants/Substitutes	No starch, cellulose, or cheaper gelling agents detectable
Functionality	Gelling agent, stabilizer, thickener, clarifier (microbiology/media uses are separate)

**Table 4:** Pros and Cons of the detection systems.

Detection System	Advantages	Insights Provided	Limitations
FTIR-ATR	Fast, non-destructive, minimal prep	Detects sulfation, uronic acids, bond breakage, new groups	Limited structural detail
NMR	Detailed structural info, no derivatization	Identifies sugar composition, linkages, substitutions	Expensive, low sensitivity, needs large pure samples
MS	Highly sensitive, detects small/rare metabolites	Identifies oligosaccharides, sulfation states, degradation products	Complex prep, matrix effects, hard to resolve isomers
Chromatography	Separates by size/charge, quantitative	Tracks degradation rate, MW distribution, supports MS/NMR	Limited structural resolution, best as complementary

## Data Availability

Not applicable.
